# Phenotypic Characterization of Patients with Polycystic Ovary Syndrome in a Population from the Ecuadorian Andes: A Cross-Sectional Study

**DOI:** 10.3390/jcm13082376

**Published:** 2024-04-19

**Authors:** María Elena Espinosa, Raúl Sánchez, Tamara Otzen, Estefanía Bautista-Valarezo, Stephanie Aguiar, Isabel Corrales-Gutierrez, Fatima Leon-Larios, Carlos Manterola

**Affiliations:** 1 PhD Program in Medical Sciences, Universidad de La Frontera, Temuco 4780000, Chile; meespinosax@utpl.edu.ec (M.E.E.); raul.sanchez@ufrontera.cl (R.S.); tamara.otzen@ufrontera.cl (T.O.); 2 Health Sciences Department, Universidad Técnica Particular de Loja, UTPL, San Cayetano alto s/n, Loja 1101608, Ecuador; mebautista@utpl.edu.ec (E.B.-V.); stephanie.aguiar@gmail.com (S.A.); 3 Center of Excellence in Translational Medicine-Scientific and Technological Bioresource (CEMT-BIOREN), Temuco 4780000, Chile; 4 Millennium Nucleus on Sociomedicine, Santiago 7560908, Chile; 5 Foetal Medicine Unit, University Hospital Virgen Macarena, 41009 Seville, Spain; icorrales@us.es; 6 Department of Surgery, Faculty of Medicine, University of Seville, 41004 Seville, Spain; 7 Nursing Department, Faculty of Nursing, Physiotherapy and Podiatry, University of Seville, 41004 Seville, Spain; fatimaleon@us.es

**Keywords:** polycystic ovary syndrome, phenotype, oligomenorrhea, hirsutism, clinical diagnosis, infertility

## Abstract

**Background:** Polycystic ovary syndrome (PCOS) is a highly prevalent endocrine–metabolic disorder in women of reproductive age. Diagnosis is based on the evidence-based international guideline 2018 and the Rotterdam Consensus to classify PCOS phenotypes. This study aims to characterize the biodemographic, clinical, metabolic, and reproductive variables and their relationship with PCOS phenotypes in a population from the Ecuadorian Andes. **Methodology:** A cross-sectional study was conducted with a non-random consecutive sample of 92 women who attended the outpatient gynecology and endocrinology clinic at the Hospital of the Technical University of Loja (UTPL)—Santa Inés, Loja, Ecuador, between January 2022 and July 2023. Descriptive statistics, mean calculations, standard deviation, parametric and nonparametric tests, odds ratios (OR), confidence intervals (CI), and *p*-values were employed. **Results:** The average age was 22 ± 3.4 years, with a predominantly mestizo, urban, single, highly educated, and medium–high socioeconomic level population. It was identified that phenotypes A + B are at a higher risk of developing oligomenorrhea and hypertriglyceridemia compared to phenotypes C + D, with statistically significant differences (*p* < 0.05). Furthermore, in terms of reproductive variables, phenotypes A + B exhibit a significantly higher frequency of elevated anti-Müllerian hormone (AMH) compared to phenotypes C + D, also with statistical significance (*p* < 0.05). **Conclusions:** The classical phenotypes A and B of PCOS are the most common in Ecuadorian Andean women and carry a higher risk of insulin resistance, anovulation, metabolic disorders, and elevated triglyceride levels compared to phenotypes C and D. Ethnic diversity and sociocultural habits influence the prevalence and clinical manifestations of these phenotypes.

## 1. Introduction

Polycystic ovary syndrome (PCOS) stands as the most prevalent endocrine metabolic disorder in women of reproductive age [1]. It affects an estimated 4–21% of women globally, constituting a leading cause of menstrual cycle disorders (MCD), female infertility (FI), and hyperandrogenism (HA) [2]. Its etiopathogenesis is intricate, multifactorial, and heterogeneous, involving genetic, epigenetic, and environmental factors, underscoring the critical importance of an accurate diagnosis and treatment [3]. Diagnostic criteria vary internationally, with the Rotterdam 2003 criteria recommended for epidemiological and phenotypic studies. A diagnosis requires the fulfillment of two out of three criteria: ovulatory dysfunction, hyperandrogenism, and polycystic ovary morphology (PCOM) [4]. Severity prediction aligns with phenotypes, and the Rotterdam consensus identifies four distinct phenotypes: A (ovulatory dysfunction + hyperandrogenism + PCOM), B (ovulatory dysfunction + hyperandrogenism), C (hyperandrogenism + PCOM), and D (ovulatory dysfunction + PCOM) [5], with phenotypes A and B being more complex [6], higher risk, and requiring prolonged treatments [7,8,9]. However, challenges persist in defining PCOS, and specific aspects of its pathophysiology remain unclear, necessitating ongoing research [10,11].

Latin America and the Andean region of Ecuador exhibit significant variability in genetic, environmental, dietary, and ethnic patterns, with limited studies characterizing PCOS phenotypes. Given diverse genetic origins, a reasonable assumption is that this genetic diversity influences PCOS phenotypic heterogeneity. Prior studies have highlighted data limitations associated with PCOS phenotypes, particularly in ethnic populations [12]. Studies in rats, such as the one by Squiccciarini et al. (2018), report that chronic cold weather influences ovarian steroidogenesis and follicular dynamics and is, therefore, likely to be a key component in the development of the polycystic ovary phenotype [13]. A systematic review indicates that the prevalence of phenotypes A and B ranges from 65.8% to 87.5% [14]. Studies in Argentina, Brazil, and Chile also identified additional complications, such as metabolic syndrome in 33.3–44% of phenotype A, 15–58% of phenotype B, 11.9–36% of phenotype C, and 14.2–66% of phenotype D, suggesting unfavorable anthropometric and metabolic profiles in Latin American women with PCOS [14]. Importantly, overweight or obese PCOS-diagnosed patients exhibit a higher prevalence of hirsutism and menstrual disorders, negatively impacting reproductive and metabolic health [15,16]. This association makes it a primary trigger for anovulatory infertility and a risk factor, increasing the likelihood of developing type 2 diabetes, insulin resistance, and cardiovascular diseases tenfold in young women—a significant public health concern and an opportunity for early intervention [17]. This study aimed to characterize biodemographic, clinical, metabolic, and reproductive variables and their association with PCOS phenotypes in an Ecuadorian Andean population, establishing a foundation for identifying the unique characteristics of these patients in future research.

## 2. Materials and Methods

### 2.1. Study Design

A cross-sectional study was developed, following the guidelines of the STROBE statement, for observational studies in epidemiology [18].

### 2.2. Sample

No sample calculation was performed because the sample corresponded to the total number of patients attended with a diagnosis of PCOS in the Hospital UTPL—Santa Inés Loja from January 2022 to July 2023. The sample was selected through non-random consecutive case sampling.

### 2.3. Data Collection and Participants

The sample selection was performed through a non-random consecutive sampling considering patients with a diagnosis of PCOS attending the gynecology and endocrinology office of the Hospital Universidad Técnica Particular de Loja (UTPL)—Santa Inés in the city of Loja (Ecuador) during the period from January 2022 to July 2023. The city of Loja is located in a region of the Andes in southern Ecuador, with an average altitude of 2200 m above sea level and a mix of ethnic backgrounds, including mestizo, indigenous, and Afro-Ecuadorian. Participants who agreed to enter the study underwent a complete medical history by health professionals, including family and personal history, and a socioeconomic survey validated by the National Institute of Statistics and Census (INEC). The women included in the study were between 18 and 40 years of age; most of the participants studied at a university center. However, teachers, administrative staff, service personnel, and private individuals also come to this healthcare center.

### 2.4. Inclusion and Exclusion Criteria

Women diagnosed with PCOS with less than one year of evolution, who were not taking contraceptives, and who met two of the three criteria described below (according to the 2018 international evidence-based guideline recommendations) were included [19].

1. Irregular cycles or ovulatory dysfunction:
  - Oligo-amenorrhea: cycles < 21 days > 35 days;
  - Less than eight menstrual cycles in a year;
  - Amenorrhea > 90 days with pregnancy previously ruled.
2. Clinical and/or biochemical hyperandrogenism:
  - Clinical data: hirsutism, androgenetic alopecia, acne (dichotomously as presence or absence);
  - Biochemical data: elevation of calculated total and free testosterone and/or other androgens (A4, DHEAS).
3. Polycystic ovarian morphology (at least in one of the two ovaries):
  - Antral follicular count ≥ 20, counting all follicles from 2 to 9 mm in each ovary in absence of follicular cyst or corpus luteum;
  - Ovarian volume > 10 mL.

Patients in gestation, puerperium, with chronic degenerative diseases (non-classical congenital adrenal hyperplasia, androgen-producing tumor, hyperprolactinemia, thyroid dysfunction, Cushing’s syndrome, drugs with androgenic activity); women with non-ovarian hyperandrogenism; and women with ovarian cysts were excluded. None of the women had received hormonal treatment at least three months before inclusion in the study. 

Notably, the 2018 and 2023 international evidence-based guidelines endorse the Rotterdam 2003 PCOS Diagnostic Criteria, requiring the presence of two of the following: (i) clinical/biochemical hyperandrogenism, (ii) ovulatory dysfunction, and (iii) polycystic ovaries on ultrasound; anti-Müllerian hormone (AMH) may alternatively be used instead of ultrasound. When irregular menstrual cycles and hyperandrogenism are present, the diagnosis is simplified, and neither ultrasound nor AMH is required for diagnosis [19,20].

**Figure 1:**
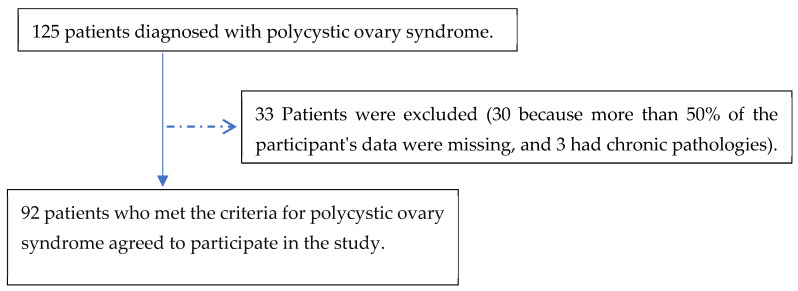
Flow diagram of the participants included in the study.

Ninety-two patients participated in the study, 85% of whom were diagnosed with PCOS in the gynecology office and 15% of whom were referred by the dermatology or endocrinology service (Figure 1).

### 2.5. Variables 

A questionnaire was applied to all participants, including the following variables: phenotypic, sociodemographic, clinical, metabolic, and reproductive.

- Phenotypic: Four clinical phenotypes of the disease have been identified, each with clinical implications regarding severity. Phenotype A is called “classical” or complete and consists of three criteria: hyperandrogenism, oligo-ovulation, and polycystic ovarian morphology. Phenotype B, also called “classic”, has hyperandrogenism and oligo-ovulation. Both phenotypes A and B have a more severe clinical and metabolic impact. Phenotype C is called “ovulatory”, characterized by hyperandrogenism and polycystic ovarian morphology, and phenotype D, “non-hyperandrogenic”, is composed of oligo-ovulation and polycystic ovarian morphology and is less severe [3].
- Sociodemographic: Age (years of age); ethnic origin (indigenous, Afro-Ecuadorian/Afro-descendant/black/mulatto/montubio/mestizo/white/other); marital status (single, married, widowed, divorced, free union); origin (urban/rural); educational level (none/early education/general primary education/high school/high school/higher education); and socioeconomic level were evaluated using the INEC survey.
- Clinical: Oligo-amenorrhea, acne, alopecia, acanthosis nigricans (presence/absence); the physical examination to determine hirsutism used the modified Ferriman–Gallwey scale (taking as normal values those above 8), which the patients described, confirmed, and evaluated by health personnel [4,21].
- Metabolic: Laboratory tests were considered extracted by a blood sample from each participant collected in vacutainer vacuum tubes without anticoagulant after fasting for 12 h taken from the antecubital vein and analyzed the same day in the hospital’s ISO 9001-certified laboratory: total cholesterol (mg/dL) ≥ 200 was abnormal; triglycerides (mg/dL) ≥ 150 was abnormal; HDL (mg/dL) ≥ 60 was abnormal; LDL (mg/dL) ≥ 160 was abnormal (based on Adult Treatment Panel III guidelines); ALT (IU/L) ≥ 33 was abnormal; AST (IU/L) ≥ 32 was abnormal; total bilirubin (mg/dL) ≥ 1.3 was abnormal; direct bilirubin (mg/dL) ≥ 0.30 was abnormal; indirect (mg/dL) ≥ 0.80 was abnormal; uric acid (mg/dL) ≥ 5.8 was abnormal; glucose (mg/dL) ≥ 126 was abnormal; HOMA-IR homeostasis assessment model ≥ 2.8 was abnormal; glycosylated hemoglobin HbA1C ≥ 6.5% was abnormal; insulin (mg/dL) ≥ 25.1 was abnormal [22].

The determination of weight and height was evaluated with a health meter (professional) scale with a measuring rod. To determine the height, the patient was asked to remove her shoes and any adornment she might be wearing on her head, to stand upright, to place her heels together, and to look straight ahead at a fixed point. With the weight and height data, the body mass index (BMI) was calculated, corresponding to the ratio between the weight expressed in kilograms and the square of the height, expressed in meters. Abdominal circumference was measured with a tape measure calibrated in millimeters and centimeters. The measurement was taken in the narrowest part of the abdomen or the region between the last rib and the navel; a value >85 cm indicates an elevated metabolic risk in the case of women. Hip circumference: Hip size was measured at the widest part of the buttocks and measured in (cm). The waist/hip index (cm) results from dividing a person’s waist circumference by his or her hip circumference, both in centimeters (cm). A value >0.86 was considered altered [22]. The waist/height index (cm) was considered abnormal when >0.49 to evaluate the distribution of body mass.

Vital signs were taken by the outpatient nursing staff (after verifying that the patient had rested for 5 min, and in case of finding altered blood pressure levels, two controls were performed at 15 and 30 min); the material for taking blood pressure was a Dash 3000 LG sphygmomanometer (CONTEC Medical Systems, Elg Grove Village, IL, USA), considering the following as abnormal: systolic blood pressure SBP (mm/Hg) ≥ 120 was abnormal; diastolic blood pressure DBP (mm/hg) ≥ 80 was abnormal. 

Other metabolic variables considered: vitamin D (ng/dL) value ≤ 20 was abnormal; ionic calcium (mmol/L) ≤ 1.1 was abnormal [23].

- Reproductive: Dehydroepiandrosterone (DHEAS) (µg/dL) ≥ 430 was abnormal; total testosterone (ng/mL) ≥ 0.482 was abnormal; free testosterone %, 17-OH progesterone(ng/mL) ≥ 1.4 was abnormal; androstenedione A4 (ng/mL) ≥ 3.9 was abnormal; free androgen index was calculated using the formula testosterone (nmol)/SHBG (nmol) × 100, considering ≥ 10% abnormal (13); anti-Müllerian hormone ≥ 2.5 was abnormal; sex cell-binding globulin SHBG (nmol/L) < 18 or ≥114.1 was abnormal; luteinizing hormone (LH) (mIU/L) ≥ 11.7 was abnormal; follicle stimulating hormone (FSH) (mIU/L) ≥ 12.5 was abnormal. Laboratory tests had to be at the follicular stage (3rd to 5th) day of menstruation in patients with amenorrhea at any period of the menstrual cycle and ultrasound after menstruation.
- Ultrasound: The ultrasound was performed by a radiologist of the Hospital UTPL—Santa Inés between the second and third days of the menstrual cycle to determine the number of antral follicles and volume. To be considered with polycystic ovarian morphology (positive), women of the sample had to have ≥20 follicles in one or both ovaries, measuring between 2 and 9 mm, and a total ovarian volume ≥ 10 cc. If a simple cyst, complex cyst, dominant follicle > 10 mm, or corpus luteum was detected, the ultrasound should be repeated in the next cycle.

The transvaginal ultrasound was performed on patients who had engaged in sexual activity, while the transabdominal ultrasound was conducted on those patients without sexual activity. In both cases, ovarian volume and cyst numbers were determined.

### 2.6. Statistical Analysis

The data obtained were entered in an Excel spreadsheet; they were analyzed with the SPSS program (IMB-SPSS, version 19.0 for Mac); and descriptive statistics were used with the calculation of frequencies, means, medians, inferential statistics, and parametric and nonparametric tests according to each variable. For the sociodemographic variables, a nonparametric test was used to compare the means of the two groups because the variable does not approach normal distribution. A logistic regression was performed to establish relationships between dichotomous variables, calculating OR values with their respective 95% CI. Values of *p* ≤ 0.05 were considered statistically significant.

### 2.7. Ethical Aspects

All subjects gave informed consent for inclusion before participating in the study. The Declaration of Helsinki conducted the study, and the protocol was approved by the Ethics Committee of the University of Cuenca: Code 2022-002EO-IE.

Each participant was assigned a code to preserve anonymity and confidentiality throughout the process. The patients who decided to participate voluntarily in the study signed an informed consent form describing the subject of the study, objectives, description of the procedures, risks and benefits, and rights of the participants, among others.

## 3. Results

Of the total of the 92 women included in this study, 61% (*n* = 56) of the patients correspond to phenotype A; 13% (*n* = 12) of the patients are phenotype B; 14% (*n* = 13) of the patients are phenotype C; and 12% (*n* = 11) of the patients are phenotype D. Due to the effects of the small sample size and the similarity of clinical characteristics, it was decided to join phenotypes A + B and phenotypes C + D to establish the different associations, resulting in the following.

### 3.1. Sociodemographic Characteristics of the Participants

The majority of the participants were under 25 years of age (%), were of mixed ethnicity (97.8%), were single (94.6%), had a high educational level (95.7%), and had a medium–high socioeconomic level (75.4%) (Table 1).

### 3.2. Clinical Features Associated with the Different PCOS Phenotypes

In women with phenotypes A and B, 97.1% experience oligo-amenorrhea, which is a striking contrast to those with phenotypes C and D. The probability of experiencing oligo-amenorrhea is 30.3 times higher in the former group, as evidenced by a statistically significant *p*-value. However, this is noted with a wide confidence interval. The variables of acne, acanthosis nigricans, and hirsutism are more frequent in phenotypes A and B compared to phenotypes C and D, but they gave no association or statistically significant value (Table 2). 

### 3.3. Metabolic Profile of the Participants

In women with phenotype A + B, 36.4% experienced hypertriglyceridemia, in contrast to the C + D phenotype. The probability of experiencing hypertriglyceridemia is 9.1 times higher in the AB group than in the CD group, with significant evidence demonstrating in their confidence interval. The BMI, waist-to-height/ratio variables, triglycerides, LDL, ALT, uric acid, insulin, blood pressure, and vitamin D have a higher percentage in the A + B phenotype than in the C + D phenotype but no statistically significant association (Table 3). 

In the metabolic biochemical evaluations, it was observed that the average HbA1c in the A + B phenotypes is (5.4), in contrast to the C + D phenotypes (5.1), with a statistically significant difference, and the average triglyceride value in the A + B phenotypes is (130.8), in contrast to the C + D phenotypes (89.7), with a statistically significant difference. For the rest of the variables, such as total cholesterol, LDL, AST, ALT, direct bilirubin, uric acid, insulin, glucose, and vitamin D, there is no statistically significant difference between the A + B phenotypes and the C + D phenotypes (Table 4).

### 3.4. Reproductive Profile of the Participants

In women with phenotypes A and B, 54.4% have elevated levels of total testosterone. Compared to those with phenotypes C and D, they are 2.4 times more likely to exhibit elevated total testosterone levels, though this difference is not statistically significant. Additionally, 16.7% of women with phenotypes A and B have an elevated free androgen index. Compared to women with phenotypes C and D, the likelihood is 3.6 times higher, but again, this finding is not statistically significant (Table 5). The variables 17 OH progesterone, LH, and AMH were also found to be higher in phenotypes A and B but did not give statistically significant values.

In the reproductive biochemical variables, it was observed that the average AMH in the A + B phenotypes is (5.9); in contrast to the C + D phenotypes (3.9), there is a statistically significant difference. For the rest of the variables, such as DHEAS, total testosterone, 17-OH progesterone, androstenedione, and FAI, there is no statistically significant difference between the A + B phenotypes and the C + D phenotypes (Table 6).

## 4. Discussion

The aim of this study was to investigate biodemographic, clinical, metabolic, and reproductive variables and their relationship with polycystic ovary syndrome phenotypes in a population of the Ecuadorian Andes. Regarding sociodemographic variables within our population, it is evident that urban origin is the most prevalent, with statistical significance (*p* < 0.05). Furthermore, phenotypes A + B are identified as being at a higher risk of developing oligomenorrhea and hypertriglyceridemia compared to phenotypes C + D, with statistically significant differences (*p* < 0.05). Additionally, regarding reproductive variables, phenotypes A + B exhibit a significantly higher frequency of elevated anti-Müllerian hormone (AMH) compared to phenotypes C + D, also with statistical significance (*p* < 0.05).

According to the last population census conducted by the Ecuadorian Institute of Statistics and Census (INEC) [24] in the province of Loja, the majority of the population is considered mestizo at 90.2%, and the average number of years of schooling for people over 24 years of age is 11.8 years and 6.5 years in the rural population. The illiteracy rate is the lowest in Ecuador at 3.2% [25].

The results of this study allow us to identify that the classical PCOS phenotypes, composed of types A and B, are the most frequent, followed by type C, “ovulatory”, and the less frequent type D, “non-hyperandrogenic”. This finding is consistent with studies from South America [21,26,27,28,29] and differs from European and Asian populations, where slightly different phenotypic patterns are observed [15,29,30].

The phenotyping of PCOS has allowed for a detailed assessment of this condition’s clinical and metabolic spectrum. Phenotypes A and B, considered classic, include a higher prevalence of oligo-amenorrhea and clinical signs of hyperandrogenism, such as acne, consistent with the previous literature [29]. In studies conducted in Latin American countries, the most prevalent phenotypes in samples with a mean age similar to our study are A and B [31], presenting as overweight and with hypertriglyceridemia, in agreement with the findings of our research. 

The association between oligo-amenorrhea and phenotypes A and B is particularly notable, reinforcing the concept that menstrual disturbance is a significant marker of classical PCOS and may be an early predictor of other metabolic and cardiovascular comorbidities [32,33]. Despite the homogeneity of participants’ sociodemographic characteristics, clinical and metabolic associations vary between phenotypes. This suggests that the underlying mechanisms leading to these manifestations may be distinct and thus require differentiated therapeutic approaches. This study, however, does not examine etiological mechanisms, which is a limitation that will need to be addressed in future research [29].

The importance of ethnicity in assessing symptoms such as acne and hirsutism was highlighted in research carried out in Brazil and El Salvador, where the Ferriman–Walley scale was applied [34]. It is essential to emphasize the need to seek standardization in the Ferriman–Walley scale modified for each of the study populations (ethnicities) for the evaluation of hirsutism [17], as sometimes the values of European populations are taken as references, which differ from those of Latin American ethnicities. It is reasonable to assume that different genetic backgrounds may influence phenotypic heterogeneity, but evidence suggests that Latin American countries have similar metabolic traits [14].

Some studies also group phenotypes A, B, and C (hyperandrogenic) and compare them with type D (non-hyperandrogenic), finding that those with hyperandrogenic PCOS have a significantly higher frequency of overweight/obesity, hyperglycemia, insulin resistance, hypertension, more altered lipid profiles, and metabolic syndrome, also associated with a higher prevalence of cardiometabolic disorders [27]. It is also reported that variations between nationalities, cultures, and ethnicities may influence elevated BMI (obesity). The visceral adiposity that is common in obese and non-obese women with PCOS worsens all metabolic and reproductive outcomes, increasing insulin resistance, compensatory hyperinsulinemia, as well as adipogenesis and decreased lipolysis. Obesity sensitizes theca cells important in LH stimulation and amplifies functional ovarian hyperandrogenism by positively regulating ovarian androgen production [35].

Previous studies have evaluated the metabolic profile of different PCOS phenotypes in Chilean and Argentinean populations, showing evidence of the impact that ethnic diversity and the socio-cultural habits of different countries may have. Hyperandrogenism may be strongly related to the development of metabolic alterations, with phenotypes A and B being those with the highest cardiovascular risk compared to phenotype D. This study reinforces the observation that phenotypes A and B have a higher risk of insulin resistance and metabolic disorders such as dyslipidemia and hepatic steatosis, with high prevalence of BMI and abnormal triglyceride levels [14]. However, high triglyceride levels have only sometimes been identified in Latin American samples [21].

In our study, we observed that especially phenotypes A and B have an elevated body mass index and waist circumference compared to phenotypes C and D. Being overweight and obese with polycystic ovary syndrome also predispose individuals to the development of a metabolic syndrome, which is not the case in lean women, with geographic differences being observed with higher risks in America compared to Europe and Asia. These differences were explained by BMI in the Americas and Asia but not in Europe, suggesting that other factors, such as genetic variation, may be involved [33]. The metabolic profile of the participants highlights the relevance of BMI and triglyceride levels in the pathogenesis of the classical phenotypes, suggesting that strategies aimed at lifestyle modification are needed to improve these parameters.

Phenotype A has been found to increase the likelihood of developing a metabolic syndrome sixfold relative to phenotype C [27], increasing its prevalence [36]. Given the age of the sample participants, which was below 25 years, it is difficult to determine the prevalence of metabolic syndromes in our sample, which is usually high in Latin American women with PCOS [14,28]. On the other hand, phenotypes C and D exhibit a milder clinical profile with a lower frequency of signs of hyperandrogenism and significant metabolic alterations, as noted in other similar studies [28]. This implies management strategies and the follow-up of patients after an individualized assessment based on phenotypes. 

As reported in a case-control study, lipid profiles are also elevated in patients with PCOS, mainly triglycerides, total cholesterol, low-density lipoprotein (LDL), and cardiovascular risk (TG/HDL). Women with PCOS type D had lower insulin levels than phenotype A [37]. In our study the triglyceride level was higher in the A phenotype. This is consistent with the results of other studies, in which phenotypes A + B are at a higher risk of having elevated triglycerides compared to phenotypes C + D, and values such as HOMA, insulin, and blood pressure levels are found to be higher in the “classical” A and B phenotypes. However, the latter were not significant in our sample.

It is described that patients diagnosed with PCOS have elevated levels of AMH and free androgen index (FAI), especially in classic phenotypes [38]. In our study, AMH exhibited statistically significant values in phenotypes A + B compared to phenotypes C + D, consistent with the literature reports. Meanwhile, the mean values for reproductive variables, such as FAI and total testosterone, approached the threshold of statistical significance.

Currently, it is recommended that serum AMH levels should not be used as an alternative for detecting the presence of polycystic ovaries (PCOM) or as a sole diagnostic test for PCOS [20]. However, it underscores the significance of tests such as elevated total or free testosterone, as well as FAI in the biochemical diagnosis of hyperandrogenism, and suggests the inclusion of examinations such as DHEAS and androstenedione for diagnosing this condition.

This study highlights the importance of identifying the prevalent PCOS phenotypes in the Ecuadorian Andean population. It emphasizes the need for an early warning and accurate diagnosis by health professionals, especially for phenotypes A and B, which present higher risks of insulin resistance and metabolic disorders. This knowledge enables the implementation of early and regular metabolic screening, helping to prevent serious long-term complications. Furthermore, it highlights the need for personalized PCOS management that considers the diversity of clinical and metabolic manifestations and integrates lifestyle interventions and specific pharmacological treatments.

Research further highlights the influence of ethnic and socio-cultural factors on the prevalence and manifestations of PCOS phenotypes, requiring that prevention and management strategies be adaptive to the cultural and social characteristics of the population. Attention to disorders such as anovulation, especially in high-risk phenotypes, and the importance of hormone monitoring, particularly of the anti-Müllerian hormone, are recognized as crucial to improving fertility management and infertility treatment planning. These findings reinforce the need for a detailed and tailored approach to the diagnosis and treatment of PCOS, considering the peculiarities of each phenotype and the specific characteristics of the patients.

The limitations include the small sample size; however, the results allow us to obtain valuable information on the phenotypic characteristics of Ecuadorian Andean women in the absence of previously published studies. We also recognize that it was impossible to carry out a control group for economic reasons since each study per patient costs 437 dollars, and private or public health insurance only covers the costs if there is evident pathology. On the other hand, it is admitted that the study is cross-sectional and does not allow for the results to be extrapolated to other populations. In addition, this study does not examine the etiological mechanisms, a limitation that should be addressed in future research. In our study, despite rigorous inclusion and exclusion criteria, the definition of variables, and the standardization of diagnostic procedures and laboratory measurements, possible biases were identified, such as selection bias, which could limit the generalizability of the findings to all PCOS patients, as the study focused on Ecuadorian women from the Andes, potentially introducing ethnic, geographic, or socio-cultural variations. Regarding reporting or classification bias, this was mitigated using the modified Ferriman–Gallwey visual scale, although self-reported symptoms, such as oligomenorrhoea or hirsutism, could lead to inaccuracies. In addition, measurement bias was considered in the ultrasound assessment to count follicles and ovarian volume, recognizing interobserver variability.

## 5. Conclusions

In this study of 92 women, 61% corresponded to polycystic ovary syndrome (PCOS) phenotype A, followed by phenotypes C (14%), B (13%), and D (12%). Phenotypes A + B and C + D were grouped due to the small sample size and similarity of clinical features. Participants, mainly young, single, and highly educated, showed significant clinical and metabolic differences between the combined groups. Women with the A + B phenotypes had a higher incidence of oligomenorrhoea, hypertriglyceridemia, and elevated HbA1c and triglyceride levels compared to the C + D phenotypes, with statistically significant differences. Although the prevalence of acne, acanthosis nigricans, hirsutism, and elevated total testosterone levels was higher in A + B, these differences were not statistically significant. This study highlights the clinical and metabolic variability within PCOS phenotypes, emphasizing the importance of a personalized approach to assessing and managing these patients.

## Figures and Tables

**Table 1:** Sociodemographic characteristics and phenotypes of PCOS.

Biodemographic Variables	Total (*n* = 92)	Phenotypes A + B (*n* = 69)	Phenotypes C + D (*n* = 23)	*p*-Value
**Age in years (mean ± SD)**	22 ± 3.4	22.6 ± 3.8	23.6 ± 5.4	0.168
**Ethnic origin (nº and %)**				
Mestiza	90 (97.8)	67 (98.5)	23 (95.8)	0.456
White	2 (2.2)	1 (1.5)	1 (4.2)	
**Origin (nº and %)**				
Urban	88 (95.7)	67 (98.5)	21 (87.5)	0.053
Rural	4 (4.3)	1 (1.5)	3 (12.5)	
**Marital status (nº and %)**				
Single	87 (94.6)	64 (94.1)	23 (95.8)	0.835
Married	4 (4.3)	3 (4.4)	1 (4.2)	
Widowed	1 (1.1)	1 (1.5)	0 (0.0)	
**Educational level (nº and %)**				
High school	4 (4.3)	3 (4.4)	1 (4.2)	0.722
Higher	88 (95.7)	65 (95.6)	23 (95.8)	
**Socioeconomic level (nº and %)**				
High	16 (23.2)	13 (25)	3 (17.6)	0.904
Medium high	36 (52.2)	27 (51.9)	9 (52.9)	
Typical medium	13 (18.8)	9 (17.3)	4 (23.5)	
Low middle	4 (5.8)	3 (5.8)	1 (5.9)	

**Table 2:** Clinical variables and association with PCOS phenotypes.

Clinical Variables	Total (%)	Phenotypes A + B *n* (%)	Phenotypes C + D *n* (%)	OR	IC 95%
Oligomenorrhea *	78 (85.7)	66 (97.1)	12 (52.2)	30.3	5.9; 153.9
Hirsutism	61 (78.2)	45 (78.9)	16 (76.2)	1.2	0.4; 3.8
Acne	74 (87.1)	56 (88.9)	18 (81.8)	1.8	0.4; 6.8
Alopecía	22 (25.3)	14 (21.2)	8 (38.1)	0.4	0.2; 1.3
Acanthosis nigricans	40 (48.2)	31 (50.0)	9 (42.9)	1.3	0.5; 3.6
Polycystic ovaries ultrasound volume ≥ 10 cc	65 (86.7)	44 (83.0)	21 (95.5)	0.2	0.1; 1.9

* Oligomenorrhoea (cycles > 35 days) or amenorrhea (no menstruation in the last three months).

**Table 3:** Metabolic variables and association with PCOS phenotypes.

Metabolic Variables	Total (%)	Phenotypes A + B *n* (%)	Phenotypes C + D *n* (%)	OR	IC 95%
BMI ≥ 25.0	29 (34.1)	24 (38.1)	5 (22.7)	2.1	0.7; 6.4
Waist-to-height/ratio ≥ 0.49	45 (60.0)	36 (63.2)	9 (50.0)	1.7	0.6; 4.9
Waist–hip/ratio ≥ 0.86	30 (40.5)	22 (40.0)	8 (42.1)	0.9	0.3; 2.6
Total Cholesterol ≥ 200 mg/dL	25 (34.2)	19 (33.9)	6 (35.3)	0.9	0.3; 2.9
Triglycerides ≥ 150 mg/dL *	21 (29.2)	20 (36.4)	1 (5.9)	9.1	1.1; 74.2
LDL ≥ 160.1 mg/dL	21 (30.0)	16 (29.6)	5 (31.3)	0.9	0.3; 3.1
ALT ≥ 33 U/L	17 (24.3)	14 (25.9)	3 (18.8)	1.4	0.3; 5.6
AST ≥ 32 U/L	11 (15.7)	8 (14.8)	3 (18.8)	0.8	0.2; 3.3
Direct bilirubin ≥ 0.30 mg/dL	6 (9.7)	4 (8.2)	2 (15.4)	0.5	0.1; 3.0
Uric acid ≥ 5.8 mg/dL	8 (10.3)	6 (10.9)	2 (8.7)	1.3	0.2; 6.7
HOMA-IR ≥ 2.8	49 (59.0)	37 (59.7)	12 (57.1)	1.1	0.4; 3.0
Insulin ≥ 25.1 uUI/mL	13 (15.5)	12 (19.0)	1 (4.8)	4.7	0.6–38.6
Blood pressure > 120/80 mm/Hg	42 (50.0)	32 (51.6)	10 (45.5)	1.3	0.5–3.4
Vitamin D < 20 ng/dL	31 (43.7)	25 (43.9)	6 (42.9)	1.1	0.3–3.4

BMI: Body Mass Index; * *p* < 0.001; LDL: Low-Density Lipoprotein; ALT: Alanine Aminotransferase; AST: Aspartate Aminotransferase; HOMA-IR: Homeostatic Model Assessment of Insulin Resistance.

**Table 4:** Biochemical evaluation of metabolic variables with the phenotypes of PCOS.

Metabolic Variables	Normal Range	Phenotypes A + B Mean (SD)	Phenotypes C + D Mean (SD)	*p*-Value
Total cholesterol (mg/dL)	50–200	189.9 (36.3)	183.3 (29.1)	0.497
Triglycerides (mg/dL)	50–200	130.8 (69.0)	89.7 (34.2)	0.021
LDL (mg/dL)	110.0–160.0	114.2 (30.1)	115.3 (34.1)	0.907
ALT (U/L)	0.0–32.0	27.2 (34.6)	24.6 (22.3)	0.776
AST (U/L)	0.0–33.0	27.8 (22.8)	26.1 (32.6)	0.813
Direct bilirubin (mg/dL)	0.0–0.30	0.2 (0.2)	0.2 (0.1)	0.668
Uric acid (mg/dL)	2.4–5.7	4.7 (0.9)	4.3 (1.3)	0.084
HOMA-IR	2.1–2.7	4.2 (3.1)	3.1 (1.9)	0.145
Insulin (uUI/mL)	2.6–25	17.3 (11.3)	12.7 (7.5)	0.093
Glucose (mg/dL)	70–115	95.3 (11.2)	91.9 (9.9)	0.199
HBA1C (%)	4.8–6.0	5.4 (0.3)	5.1 (0.2)	0.001
Vitamin D (ng/dL)	20–160	26.1 (15.3)	26.7 (22.1)	0.900

LDL: Low-Density Lipoprotein; ALT: Alanine Aminotransferase; AST: Aspartate Aminotransferase; HOMA-IR: Homeostatic Model Assessment of Insulin Resistance.

**Table 5:** Reproductive variables and association with PCOS phenotypes.

Reproductive Variables	Total (%)	Phenotypes A + B (%)	Phenotypes C + D (%)	OR	IC 95%
DHEAS ≥ 430 µg/dL	6 (7.7)	5 (8.3)	1 (5.6)	1.5	0.2–14.2
Total testosterone ≥ 0.482 ng/mL	45 (48.9)	37 (54.4)	8 (33.3)	2.4	0.9–6.3
17-OH progesterone ≥ 1.4 ng/mL	37 (51.4)	32 (57.1)	5 (31.3)	2.9	0.9–9.6
Androstenedione ≥ 3.9 ng/mL	22 (29.3)	17 (29.3)	5 (29.4)	1.0	0.3–3.2
LH (follicular phase) ≥ 11.7 mUI/mL	30 (40.0)	25 (44.6)	5 (26.3)	2.3	0.7–7.1
SHBG < 18 nmol/L	14 (16.3)	11 (17.2)	3 (13.6)	0.8	0.2–3.0
Free androgen index(FAI) ≥ 10%	11 (13.9)	10 (16.7)	1 (5.3)	3.6	0.4–30.1
AMH ≥ 2.5 ng/mL	51 (91.1)	41 (93.2)	10 (83.3)	3.4	0.2–65.8

DHEAS: Dehydroepiandrosterone Sulfate; LH: Luteinizing Hormone; SHBG: Sex Hormone-Binding Globulin; AMH: Anti-Müllerian Hormone.

**Table 6:** Biochemical evaluation of reproductive variables with the phenotypes of PCOS.

Reproductive Variables	Normal Range	Phenotypes A + B Mean (SD)	Phenotypes C + D Mean (SD)	*p*-Value
DHEAS (µg/dL)	35.0–430.0	262.9 (217.5)	243.9 (141.9)	0.727
Total testosterone (ng/mL)	0.084–0.481	0.5 (0.2)	0.4 (0.2)	0.062
17-OH progesterone (ng/mL)	0.2–1.3	1.7 (1.1)	1.5 (1.3)	0.497
Androstenedione (ng/mL)	0.3–3.3	3.2 (1.4)	3.1 (1.6)	0.983
LH (follicular phase) (mUI/mL)	1.1–11.6	12.5 (9.2)	9.7(3.9)	0.196
FSH (follicular phase) (mUI/mL)	3.50–12.50	4.9 (2.2)	5.1 (1.9)	0.808
SHBG (nmol/L)	18.0–114.0	73.1 (66.5)	73.8 (81.8)	0.970
Free androgen index (FAI) %	5–10	3.2 (2.9)	1.9 (1.4)	0.070
AMH (ng/mL)	1.22–11.27	5.9 (3.2)	3.9 (1.5)	0.045

DHEAS: Dehydroepiandrosterone Sulfate; LH: Luteinizing Hormone; FSH: Follicle-Stimulating Hormone; SHBG: Sex Hormone-Binding Globulin; AMH: Anti-Müllerian Hormone.

## Data Availability

Data is unavailable due to privacy.
